# Environment Changes, Aflatoxins, and Health Issues, a Review

**DOI:** 10.3390/ijerph17217850

**Published:** 2020-10-27

**Authors:** Rafael Valencia-Quintana, Mirta Milić, Daniela Jakšić, Maja Šegvić Klarić, María Guadalupe Tenorio-Arvide, Guillermo Alejandro Pérez-Flores, Stefano Bonassi, Juana Sánchez-Alarcón

**Affiliations:** 1 Facultad de Agrobiología, Universidad Autónoma de Tlaxcala, Tlaxcala 90120, Mexico; prvq2004@yahoo.com.mx (R.V.-Q.); gaperezf@gmail.com (G.A.P.-F.); 2 Mutagenesis Unit, Institute for Medical Research and Occupational Health, Ksaverska cesta 2, 10000 Zagreb, Croatia; mmilic@imi.hr; 3 Department of Microbiology, Faculty of Pharmacy and Biochemistry, University of Zagreb, Schrottova 39, 10000 Zagreb, Croatia; djaksic@pharma.hr (D.J.); msegvic@pharma.hr (M.Š.K.); 4 Departamento de Investigación en Ciencias Agrícolas, Benemérita Universidad Autónoma de Puebla, Puebla 72570, Mexico; 5 Department of Human Sciences and Quality of Life Promotion, San Raffaele University, 00166 Rome, Italy; stefano.bonassi@sanraffaele.it; 6 Unit of Clinical and Molecular Epidemiology IRCCS San Raffaele Pisana, 00166 Rome, Italy

**Keywords:** climate change, AFB_1_, *Aspergillus flavus*, crops contamination, mycotoxins

## Abstract

Crops contaminated by aflatoxins (AFs), the toxic and carcinogenic mycotoxins produced namely by *Aspergillus flavus* and *Aspergillus parasiticus*, have severe impacts on human health. Changes in temperature and water availability related to actual climate changes (increased temperature, heavy rainfalls, and droughts) are modulating factors of mould growth and production of mycotoxins. To protect human and animal health from the harmful effects caused by AFs, the development of a safe and effective multifaceted approach in combating food and feed contamination with AFs is necessary. This review aims to collect and analyze the available information regarding AF presence in food and feed to reinforce AF management and to prevent health issues related to the AF exposure in the light of actual climate changes.

## 1. Introduction

Climate is the key factor that drives fungal colonization and mycotoxin production [1]. Consequently, the observed climate changes are complex, multifaceted and interconnected, resulting in a serious impairment of the availability of food and feed in developing countries, particularly in terms of food security [2,3]. Hence, predicting the effects of climate change on where, which, and by how much mycotoxins are going to change is of utmost importance [4]. Among thousands of secondary metabolites produced by agriculturally important filamentous fungi, there are several groups of mycotoxins with worldwide distribution and of special interest: aflatoxins (AFs), deoxynivalenol (DON), fumonisins (FUMs), zearalenone (ZEA), and ochratoxin A (OTA). They represent an unavoidable problem due to their presence in globally consumed cereals such as rice, maize, and wheat, but also other vegetables and fruits. Variety of toxic properties are described for mycotoxins and among them there are carcinogens, hepatotoxins, nephrotoxins, neurotoxins, and many of them are capable of different immunomodulatory effects. Because of their global distribution edging with inevitability in food/feed and the toxicity of these compounds, worldwide trends envision a stricter control of mycotoxins. However, the changing global environment may not be the ideal setting to control and reduce the exposure to these toxins [5]. Production of these compounds is highly susceptible to environmental factors, pre- and/or post-harvest, thus, when changes in the weather occur, mycotoxins will be affected [4]. The situation is even more complicated by the fact that contamination also occurs during storage and transportation because different indoor environments are recognised sources of both fungal producers and their metabolites [6,7,8,9,10].

Among all mycotoxins, the biggest attention has been granted to AFs, namely AFB_1_ because it is a well-known human carcinogen (group 1, IARC, International Agency for Research on Cancer). The main producers of AFB_1_ are *Aspergilli* from the section *Flavi*. Although these fungal species are distributed worldwide, AFs producers were always more prevalent in tropical and subtropical regions. Based on recent studies, AFs produce fungi and consequently AFs are becoming more prevalent in countries with temperate climate [3,10,11,12,13,14].

In the light of changing weather conditions, it is necessary to continuously monitor the presence of AFs producers in food and feed matrices but also in the indoor environments where the food/feed is stored. These results could be useful for developing a climate prediction model that could add to improvement in agriculture. Consequently, it might contribute to human health by preventing deleterious effects related to ingestion of contaminated food and nonetheless, food and feed safe for consumption add to mental and social well-being in terms of economic success.

## 2. Aflatoxins: Producers, Distribution, and Related Health Issues

The main producers of AFB_1_ belong to the *Aspergillus* section *Flavi*, phylogenetically comprised of 33 species. Among these, *A. pseudotamarii* and *A. togoensis* only produce B-type AFs, AFB_1_ and B_2_ [15]. While it is generally accepted that *A. flavus* only produces B-types AFs and is unable to produce G- types AFs, it was reported that some Korean strains also produce AFG_1_ and G_2_ [15]. Many species are able to produce B- and G- types of AFs (AFB_1_, AFB_2_, AFG_1_ and AFG_2_) like *A. cerealis, A. aflatoxiformans, A. arachidicola, A. austwickii, A. luteovirescens, A. minisclerotigenes, A. mottae, A. nomius, A. novoparasiticus, A. parasiticus, A. pseudocaelatus, A. pseudonomius, A. sergii* and *A. transmontanensis* [15]. Other *Aspergilli* able to produce AFs are the species from the section *Nidulantes* (*A. astellatus, A. olivicola* and *A. venezuelensis*) and section *Ochraceorosei* (*A. ochraceoroseus* and *A. rambellii*) [16]. In addition, AFs are also produced by insect pathogens *Aschersonia coffea* and *Aschersonia marginata* [17,18].

*A. flavus* was first recognized to cause aflatoxicosis in domestic animals, causing the death of thousands of turkeys back in 1960 [19]. The event triggered the monitoring of AFs in different food/feed commodities including corn, peanuts, and rice. The second important producer of AFs, *A. parasiticus*, is primarily associated with peanuts in the Americas but also can occur on corn, figs, and pistachios [20]. Other structural analogues of AFs were identified like P_1_, Q_1_, B_2a_, G_2a_, D_i_, B_3_ and M_1_ that may occur in different foods following mammalian biotransformation [13]. Although AFM_1_ has been mainly related to milk as a product of AFB_1_ liver metabolism in animals, recently it was described as a product of *A. flavus* biosynthesis [21,22] and therefore it could be expected in contaminated foodstuff.

*A. flavus* has a wide range of temperature tolerance (19–35 °C) with about 28 °C optimum for growth and 28–30 °C for AFs production [4,23]. Water activity (a_w_) or free water in the substrate, e.g., host tissue, is another essential factor for fungal growth and mycotoxins production. Some strains of *A. flavus* can grow at very low a_w_ of 0.73 while AFs are optimally produced at a_w_ 0.85 [24]. Therefore, drought-, nutrient-, or temperature-stressed maize or peanut plants are more susceptible to colonization by *A. flavus* or *A. parasiticus* [25,26,27,28]. Sudden changes in rainfall/drought patterns and a consecutive humidity in addition to temperature and CO_2_ increase directly affects expression of regulatory (aflR) and structural genes (aflD) involved in AFs biosynthesis [29].

AFB_1_, the most toxic of the AFs, is probably the most thoroughly studied of all mycotoxins and there are many detailed reviews regarding its toxicology [5,30,31,32,33,34,35,36,37]. Briefly put, it acts as immune-toxic and hepatotoxic, it contributes to an impaired productivity and reproductive efficiency, and it promotes inflammation and acts as a growth suppressor [38]. Being associated with approximately 25% of liver cancer globally, or 172,000 liver cancers per year [39], AFB_1_ is the most potent naturally occurring liver carcinogen known. Additionally, there is a highly worrisome link between AFB_1_ exposure and childhood stunting, which can lead to a lasting variety of adverse health conditions [40].

## 3. Legislation of AFs

The toxicity of AFs is recognized by the Joint Expert Committee on Food Additives (JECFA, scientific advisory board of Food and Agriculture Organization, FAO, and World Health Organization, WHO) and regarding the threat to human health, legal regulatory limits have been established [41,42]. In their concluding remarks on JECFA’s eighty-third meeting regarding the AFs maximum limit in peanuts held in Rome in 2016, the board supported an increase of maximum limit of AFs in peanuts to 15 µg/kg as it would double the availability of ready-to-eat-peanuts on the world market while at the same time these concentrations are not expected to impact the exposure of the general population to AFs [42]. However, based on a scientific opinion of the Panel on Contaminants in the Food Chain (CONTAM Panel) the calculated cancer risk would increase by a factor of 1.6–1.8 if the maximum level of AFs were increased from 4 to 10 µg/kg in peanuts and related products intended for human consumption [43]. However, the panel recommended a full risk assessment to be carried out in the future in which the observed elevated AFs levels in food should be considered [43]. Regarding these limits, there are several problems that can be noticed: the limits are not harmonized among the countries; the regulation does not apply to the same food/food products and the limits apply to AFB_1_ and/or total AFs. In EU particularly, the content of AFB_1_ is limited to 2 µg/kg in food intended for direct consumption and 5 µg/kg if it is intended for further processing; in Australia the regulatory limit is 15 µg/kg and applies to total AFB_1_ in peanuts and tree nuts; in USA AFB_1_ in all food crops is limited to 20 µg/kg while in Brazil to 30 µg/kg; in Japan AFB_1_ is limited to 10 µg/kg [41,44]. One of the most frequently and heavily contaminated food products are pistachios where the estimated mean concentration of AFs in time of the last comprehensive evaluation was 54 µg/kg [41]. Considering those data, the proportion of rejected samples based on the legal regulatory limits is expected to be 40%–60%. Similar projections were made to other nuts, dried fruit, and maize. The conclusion that can be drawn out is that the food products (different nuts, spices, cocoa, dried fruits, etc.) from South America and Middle East often contain AFB_1_/AFs; the importing countries, especially EU, have a way stricter regulatory limits than the exporting countries. This reflects on an increased price of the products (importing countries) and a higher exposure to AFB_1_/AFs (exporting countries). The data from a study in Algeria showed that *A. flavus* was the most prevalent species isolated from wheat. Among *A. flavus* isolates, 72% produced AFB_1_ in rather high concentrations (12.1 to 234.6 µg/g of CYA medium), while AFB_1_ was detected in 56% of wheat and wheat products in concentrations ranging from 0.13 to 37.42 µg/kg [45]. Additionally, in a study conducted in Pakistan, concentrations of aflatoxins detected in rice, wheat, maize, barley, sorghum, red kidney beans, split peas, and soybeans exceeded the maximum concentrations of total aflatoxins set by the EU [46]. The highest contamination levels of AFs were found in a sample of wheat (15.5 µg/kg), maize (13.0 µg/kg), and barley (12.6 µg/kg) [46]. Prediction scenarios based on expected temperature increase +2 °C and +5 °C in Europe, suggesting an increase in contamination of maize with *A. flavus* while under the same conditions it is not expected for other crops, namely wheat and rice [47]. However, the situation in warmer climates may differ. In the latest risk assessment of AFs conducted by EFSA, it was concluded that pistachios, peanuts, other legumes, and seeds should be continuously monitored as food where the contamination with AFB_1_ and total AFs is expected [48]. In some Asian countries the peanuts are an important part of everyday diet and regulatory actions are continuously employed in order to avoid excessive consumer exposure. Considering the margins of exposure limits (MOE) used as a tool to improve food safety management, levels of AFs in peanuts and peanut products that were imported to Taiwan from China, Indonesia, Thailand, the United States, and the Philippines were above the safe lower limit of 10,000, while the products from Vietnam were under the MOE safe lower limit [49].

In the light of a global changing environment, it may not be enough to employ sensitive analytical methods that enable precise AFs detection and quantification. The attention should be paid to any food/food products where the contamination of aflatoxigenic strains is expected because of the potential of proliferation and AFs production if favorable conditions occur. Additionally, an important thing to consider is a change in ecology of AFs-producing fungi in food-importing countries because, in theory, global warming, altered CO_2_, and humidity patterns may support proliferation of fungal companions of imported food. Several years ago, *A. nomius* and *A. pseudonomius* were detected for the first time in food samples from Central and Eastern Europe and they were able to produce B and G types of AFs [50]. Previously, *A. pseudonomius* was detected in insects and soil samples from the USA [51] and house dust samples in Thailand and Micronesia [52] and it contributed to aflatoxin contamination of Brazil nuts in Brazil [53]. 

## 4. The Problem: Climate Change and Mycotoxins 

Climate changes have been observed for decades now. Uncertainties in air temperature and rainfall patterns represent special challenges in food production and processing in developing countries [54]. Paterson and Lima anticipated several challenges for those countries since 2010, especially those with temperate climates, since the climate in these regions is becoming warmer (about 33 °C), close to the optimal aflatoxin production, for example. However, the climate conditions will be not the only concern, since Moretti and collaborates assume new combinations of mycotoxins/host plants/geographical areas; in order to understand the mycotoxin contamination will require experience of the scientific community focused on toxigenic fungi [55]. Grains are highly represented in the human diet evidenced by global production of more than 2790 million metric tons in 2020 [56]. Based on a study conducted by FAO, approximately 25% of crops are affected by mycotoxins [55]. Original reference and validity of this percentage was recently questioned and inspected by Eskola and co-authors [44]. They pointed that 25% estimated by FAO correlates with the values above EU and Codex limits for samples collected at the source, while it underestimates the overall presence of mycotoxins [44]. It was estimated that climate change might affect economic losses in the maize industry in the USA of up to $1.7 billion due to AF contamination [57]. The problem of mycotoxins in food in the era of climate change awareness is particularly challenging. The data about the mycotoxins in food may be scarce due to lack of adequate analytical methods and data collection practices, while emerging mycotoxins add to the complexity of the whole picture. As global warming awareness started to rise back in the 1970s, it was obvious that environmental issues demanded a multinational approach, particularly to meet the need for standardized global research programs. An important milestone in the history of global warming awareness was the establishment of the Intergovernmental Panel on Climate Change (IPCC), a unique hybrid of a scientific and a political body. In their first report issued in 1990, it was brought to attention that the Earth is warming with the expected increase in average temperature between 1.5 and 4.5 °C by 2050. Since then, on every meeting of the panel the decrease of CO_2_ release was highly demanded. The threats of climate change are recognized by many political and civil organizations including the United Nations as the delegates urged the international community to reduce greenhouse gas emissions, conserve forests, and monitor water sources [58]. The strategies in combating climate change differ among the developed countries and developing countries. In developing countries like Sri Lanka, Guatemala, Yemen, Liberia, Brazil, South Africa, Indonesia, Iran, India and many other members of the Group of 77 (G77) at the United Nations, a resolution of many socioeconomic issues like poverty, hunger, unemployment rates, coups against legitimate governments, civil wars and lack of education, illicit financial flows and other [58] are prerequisites to a successful food safety management. In economically stable and more developed countries it may be easier to employ sophisticated methods that enable reliable identification of mycotoxin-producing fungi, detection of mycotoxins, and advanced data management. Nonetheless, technologies required for advanced agricultural planning, food production, and long-term storage facilities resistant to natural climate conditions and natural contaminants also require high socioeconomic standards of the country in which they are intended to employ. However, even in developed countries, about 4.5 billion people are exposed to uncontrolled and unmonitored amounts of mycotoxins, mostly due to import of contaminated food [5].

## 5. The Impact of Environmental Factors on AFs

Water availability, temperature, and their interactions through precipitation patterns and extreme weather events as floods and droughts impact fungal growth, proliferation, and secondary metabolism [59,60,61], in addition to untimely harvest time, improper drying, and poor storage. To understand the impact of climate changes on mycotoxins production it is important to understand the optimal conditions for fungal growth and the circumstances under which the mycotoxins are produced. Mycotoxins are small molecules produced during morphological and chemical differentiation of fungi. They are exometabolites which means they are secreted or deposited in or on the cell wall and accumulated. Although different species may produce the same mycotoxin, the exometabolom is taxonomically restricted because the exometabolites are produced in species-specific profiles [62]. In addition, association of mycotoxins-producing fungi to certain plants and animals have contributed to complexity and evolution of secondary metabolism [62]. While studying the growth and mycotoxin-producing abilities of important fungal food contaminants, solid media like potato dextrose agar, malt extract agar, and Czapek yeast extract agar are most commonly used [9,63]. However, it was brought to attention by some authors that semi-synthetic growth media, e.g., maize- and peanut-based, represent the better alternative in ecophysiological studies of important food pathogens like *A. flavus* [64]. Mathematical models are useful tools in understanding the role of water availability, pH, and temperature on fungal growth and mycotoxin-producing abilities. For example, Baranyi’s model and other potentially suitable secondary models, Rosso, polynomial and Davey, were employed to describe the radial growth rate of *A. flavus* on brown rice as a function of temperature and a_w_ [65]. In addition to essential factors for fungal growth, i.e., temperature, a_w_ and pH, a Box-Behnken design was used to inspect the effect of protein concentration, fat, and essential oil on the growth of *A. flavus* during 50 days of incubation while the fungal response was modeled by the modified Gompertz equation, logistic regression, and time-to-detection model. [66]. These predictive models may be useful tools in accurate prediction of the probability or the time at which *A. flavus* growth occurs in the field or under storage conditions. Both temperature and humidity influence the fungal damage of the crops. Warm conditions favor the growth of AFs-producers, thus global warming poses a potential problem in this regard, particularly in currently temperate climates [4]. Proposed mechanism for increased AFs biosynthesis under influence of temperature, a_w_ and CO_2_ is modulation of expression of key regulatory genes *aflR* and *aflS* in AFs biosynthesis cluster [67,68,69] and structural genes, i.e., norsolorinic reductase coding *aflD, aflE and aflF* [57,70,71]. Additionally, it is important to consider the influences of temperature, a_w_, and CO_2_ on the substrate, i.e., plant, by interfering with carbon absorption or oxygen depletion [72].

Droughts are another important trigger of AFs biosynthesis, as observed in tropical countries, where acute aflatoxicosis upon ingestion of poor crops is often reported [73]. Central Europe suffered damaging drought records in 2018 and 2019, with record summer temperatures measured on the continent. If global greenhouse gas emissions rise strongly, the extreme droughts are expected in central Europe up to seven times more often with the drastic consequences in agriculture [74]. Witnessed year-to-year unpredicted weather conditions are making any available weather-based food control strategies difficult to reliably employ. Understanding the relationship between environmental stress and the production of secondary metabolites at the molecular and transcriptomic level is paramount to rational crop protection approaches and sustainable food production planning. 

As a future perspective, there are “space technologies” that have already found application in public health strategies. Remote sensing (data collection at distance, usually from a satellite or an aircraft as opposed to on-site sensing), global navigation satellite systems (satellite ensembles that allow any user on or near the Earth to determine their position with a precision from some meters to some centimeters, e.g., GPS), and others were recognized as valuable benefits to global health and their applications were recently reviewed [75]. Such technologies were used in direct monitoring of certain microorganisms as well as identifying associations between diseases and/or disease vectors and remotely sensed parameters, e.g., malaria or tick-borne diseases and rainfall, vegetation indices, and temperature [76,77]. Those associations enabled forecasting of the spatiotemporal evolution of diseases and preparation of rational public health strategies. Remote sensing techniques were exploited in monitoring concentrations of some toxins with the distinct fluorescent properties, like those derived from cyanobacteria [78]. Similarly, it could be used to understand possible effects of climate change on mycotoxin contamination and in monitoring of AFs and AFs-producing fungi.

## 6. Conclusions

Climate change inevitably affects agriculture, mould colonization patterns, and excretion of various secondary metabolites, including AFs. It implies the necessity for developing models for the prediction of future scenarios and strategies for safe and sufficient agriculture-based food production. The observed changes are not expected to influence all species of mycotoxigenic fungi in the same way because of competitiveness of fungi that adds to selection. 

In the light of climate change, there is a need for intersectorial and international cooperation to improve and maintain food safety through harmonized monitoring strategies and regulation limits that should be developed and implemented into the global system.
